# Combining tissue-derived microRNAs with clinical risk models for prediction of HCC recurrence after liver transplantation: A proof-of-concept study

**DOI:** 10.1038/s41598-026-41688-9

**Published:** 2026-02-27

**Authors:** Theresa Lederer, Konrad Lehr, Stefanie Bobe, Kim Falkenberg, Cosima Thon, Emily Hoffmann, Wolfgang Roll, Florian Rennebaum, Haluk Morgül, Max Masthoff, Osman Öcal, Jonel Trebicka, Moritz Wildgruber, Alexander Link, Philipp Schindler

**Affiliations:** 1 Department of Gastroenterology, Hepatology and Infectious Diseases, Otto-von-Guericke University Magdeburg, Magdeburg, Germany; 2 Gerhard Domagk Institute of Pathology, University of Münster, Münster, Germany; 3 European Institute for Molecular Imaging, University of Münster, Münster, Germany; 4 Department of Radiology, University of Münster, Münster, Germany; 5 Department of Nuclear Medicine, University of Münster, Münster, Germany; 6 Department of Internal Medicine B, University of Münster, Münster, Germany; 7 Department of General, Visceral and Transplant Surgery, University of Münster, Münster, Germany; 8 Clinic for Diagnostic and Interventional Radiology, University of Heidelberg, Heidelberg, Germany; 9 Department of Radiology, LMU Munich, Munich, Germany; 10 Department of Gastroenterology, University of Erlangen-Nuremberg, Medical Campus Upper Franconia, Klinikum Bayreuth, Bayreuth, Germany

**Keywords:** HCC, Liver transplantation, MiRNA, Integrated diagnostics, Precision oncology, Biomarkers, Cancer, Computational biology and bioinformatics, Gastroenterology, Oncology

## Abstract

To evaluate the utility of microRNAs (miRNAs) integrated with current clinical risk models as predictive models for hepatocellular carcinoma (HCC) recurrence after liver transplantation (LT). This retrospective proof-of-concept study included 20 patients with HCC who underwent LT between 2007 and 2021 (*n* = 10 recurrent, *n* = 10 5-year recurrence-free). MiRNA profiling was performed on formalin-fixed, paraffin-embedded (FFPE) HCC explant tissue at the time of transplantation and clinical data were collected. The predictive value of miRNA expression for HCC recurrence was evaluated in a hybrid data- and hypothesis-driven approach and combined with clinical risk models (Milan, UCSF, Metroticket 2.0 and AFP). Kaplan-Meier analysis was performed to analyze recurrence-free survival (RFS). We identified a 3-miRNA signature - miR-3692-5p, miR-424, and miR-718 - that revealed discriminatory capacity between recurrence and non-recurrence. Adding this signature to clinical models increased the area under the receiver operating characteristic curve (AUC) for modeling HCC recurrence from 0.5 to 0.7 to 0.94–0.96. The combined models were used to categorize patients as high- or low-risk, with patients in the high-risk group having a shorter estimated median RFS (17.0 months vs. 38.5 months, *p* < 0.05). Integrating tissue-derived molecular miRNA signatures with existing clinical risk models may enhance the prediction of HCC recurrence following LT. Incorporating molecular approaches into current protocols could refine post-transplant risk stratification and surveillance guidance.

## Introduction

Hepatocellular carcinoma (HCC) the most common primary malignant liver tumor worldwide and remains a leading cause of cancer-related mortality^1,2^. For patients with early unresectable HCC, liver transplantation (LT) is the first-line treatment of choice; however, limited donor organ availability and post-transplant recurrence remain critical concerns and place special demands on patient selection^3,4^.

The Milan criteria, introduced in the 1990 s, have been widely adopted as the standard for selecting HCC patients for liver transplantation, emphasizing tumor size and number^5^. Alternative, more extended criteria such as UCSF or up-to-7 also focus on the size and number of HCC lesions and have been shown to be non-inferior to the Milan criteria in terms of post-transplant survival^6–8^. However, even with the adoption of restrictive selection criteria, post-transplant HCC recurrence rates currently range from 8 to 20%^9,10^. There is increasing evidence that these criteria fail to capture the biological behavior of HCC, such as tumor grade or microscopic vascular invasion, as prognostic factors for disease recurrence and transplant failure^11–13^.

To address these challenges, advanced clinical and imaging risk scores are emerging that go beyond the traditional Milan criteria and incorporate various factors ranging from Alpha-fetoprotein (AFP) levels to neutrophil-to-lymphocyte ratios to better predict HCC behavior and risk of recurrence^12,14–17^. An even more comprehensive strategy to refine patient management and improve post-transplant surveillance may be provided by the future incorporation of molecular markers into these criteria, as recommended by current guidelines^18,19^. Within this concept, microRNAs (miRNAs) are considered as promising molecular markers.

MicroRNAs (miRNAs) are small, non-coding RNA molecules involved in post-transcriptional gene regulation that have attracted considerable interest as potential biomarkers in a variety of malignancies, including HCC^20–22^. In particular, tissue-derived miRNAs offer distinct advantages for disease monitoring due to their stability and tissue-specific expression patterns^23^. Accumulating evidence suggests that certain miRNA signatures may correlate with tumor aggressiveness and clinical outcomes, making them promising tools for refining risk stratification^24–27^.

Therefore, this proof-of-concept study is hypothesis-generating and aims to combine tissue-derived miRNA profiles with current clinical risk models to propose a more refined and personalized post-transplant risk assessment and surveillance for HCC patients.

## Materials and methods

### Ethics approval and compliance with guidelines

This retrospective study was approved by the local Institutional Review Board (Ethics Committee of Westphalia-Lippe, ID: 2022-170-F-S, approval date: May 19, 2022) and was conducted at an academic liver transplant center. All methods were performed in accordance with relevant guidelines and regulations, including the Declaration of Helsinki and institutional protocols. Due to the retrospective nature of the study, informed consent was waived. Patient data derived from electronic medical records were pseudonymized prior to analysis to ensure confidentiality. No organs or tissues were procured from prisoners. All liver transplantations were performed at the Department of General, Visceral, and Transplant Surgery at the University of Münster in Münster, Germany, under established clinical protocols. According to the Istanbul Declaration, all patients had previously given informed consent for LT and for organ allocation according to the Eurotransplant rescue allocation protocols.

### Patient selection

Selection criteria for LT were defined by the center’s standard operating procedures and excluded individuals with extrahepatic tumor spread or macrovascular invasion. Treatment decisions were based on tumor burden (size and number of lesions) without a strict upper limit for eligibility. All eligible patients received neoadjuvant locoregional or systemic bridging/downstaging therapies, with response (CR, complete response; PR, partial response) assessed using the modified Response Evaluation Criteria in Solid Tumors (mRECIST)^28^. Initially, patients who met the Milan criteria were eligible for standard exception points, while those who exceeded the criteria could be considered if they achieved stable or responsive disease over at least six months of neoadjuvant therapy. Such patients generally received grafts from extended criteria donors, as they were not prioritized on the waiting list.

Patients from the entire cohort with HCC who underwent LT between 2007 and 2021 (*n* = 116) were reviewed if they met the following criteria: (1) age ≥ 18 years, (2) diagnosis of HCC confirmed by histopathology, (3) availability of a diagnostic CT scan within six months before LT (for patients without preoperative treatment) or a baseline CT scan before initiation of bridging therapy, (4) no history of other malignancies and (5) archived formalin-fixed paraffin-embedded (FFPE) HCC tumor tissue obtained from liver explants after completion of clinical diagnosis. Exclusion criteria were mixed HCC-cholangiocarcinoma, insufficient specimen quality for analysis, missing arterial or portal venous phase imaging, or less than one month of follow-up after LT.

Of all eligible patients (*n* = 81), *n* = 20 were randomized for this proof-of-concept approach. A computer-generated randomization list was generated and stratified by HCC recurrence or 5-year recurrence-free, using a block size of 10.

### Data collection and follow-up

Demographic, clinical, and procedural data, including sex, age at LT, etiology of liver disease, laboratory parameters, waiting list duration, bridging therapies, and follow-up/survival information, were collected retrospectively from electronic medical records and the picture archiving and communication system (PACS). During the first two years post-LT, patients underwent surveillance every 3–6 months, which included measurement of alpha-fetoprotein (AFP) levels and ultrasound examinations. Beyond two years post-transplant, follow-up visits and imaging studies were typically scheduled at annual intervals.

The primary endpoint was HCC recurrence after LT, defined radiologically according to EASL criteria^18^ and/or confirmed histopathologically. Recurrence-free survival (RFS) was measured from the date of LT to the date of documented HCC recurrence - either intrahepatic, extrahepatic metastasis or death from any cause, whichever occurred first. For patients without recurrence or death by the data cut-off date (March 1, 2023), RFS was censored at the date of the most recent appropriate disease assessment. Patients were stratified according to confirmed HCC recurrence after LT or 5-year recurrence-free survival (non-recurrence).

### Imaging analysis

All patients underwent contrast-enhanced CT of the liver. The imaging protocol consisted of arterial phase images acquired 20–35 s after contrast administration and portal venous phase images acquired at 60–70 s. Two abdominal radiologists with eight years of experience in liver imaging independently reviewed the CT images without access to clinical or laboratory data. All lesions that met the radiological criteria for HCC (equivalent to LI-RADS 4 and 5) were included in the analysis^29^. Lesion size and number (≥ 1 cm) were recorded.

### RNA isolation

Following the completion of the full diagnostic work-up, H&E-stained sections were reviewed to identify a suitable sample for RNA isolation. For each patient, a single FFPE tissue block containing a representative area of HCC tissue was selected. The HCC region within this block was then dissected, and RNA was extracted from 5 μm thick sections using the Qiagen EZ2 RNA FFPE kit (Qiagen, Hilden, Germany), following the manufacturer’s instructions. The isolated RNA was stored at −80 °C until further miRNA processing.

### MiRNA isolation and microarray analysis

Total miRNAs were extracted according to the previously described protocol with the miRNeasy Mini Kit, following the instructions provided by the manufacturer (Qiagen, Hilden, Germany)^30^. The concentration and quality of miRNAs were determined photometrically with *NanoDrop* (ThermoFisher Scientific, Darmstadt, Germany). The microarray experiment was carried out using Affymetrix Gene Chip Microarray technology (Gene Chip Microarray 4.0, Affymetrix, Santa Clara, USA) according to the manufacturer’s instructions. The analysis was performed, further analyzed and normalized individually for each cohort using the *Transcriptome Analysis Console* (TAC). Only human miRNAs were included in the analyses.

### Study design

**Fig. 1:**
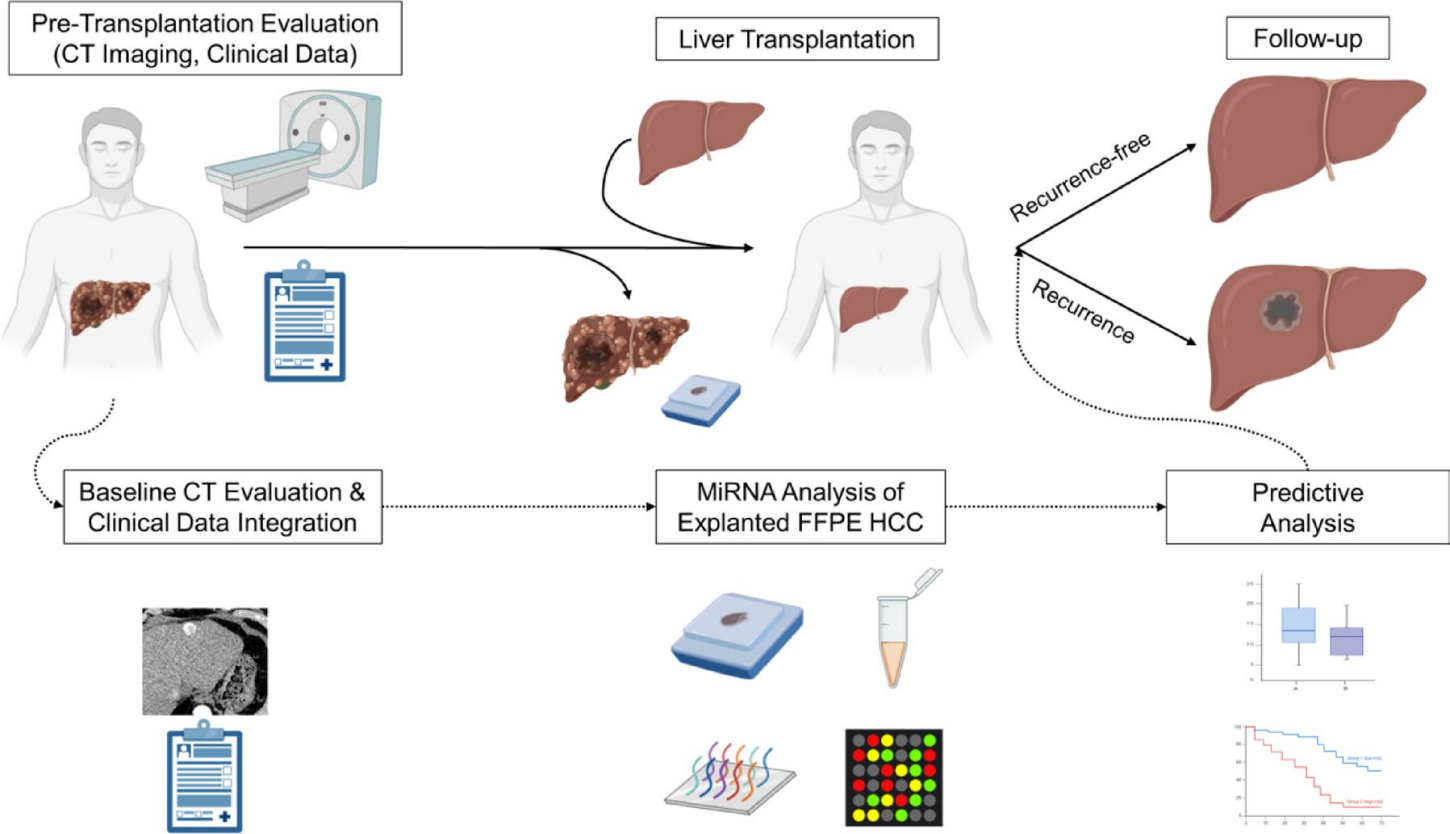
Study design. Abbreviations: CT, Computed tomography; FFPE, Formalin-fixed, paraffin-embedded; HCC, Hepatocellular carcinoma; MiRNA, MicroRNA.

To analyze the added value of tissue-derived miRNA profiles with current clinical risk models, the following hybrid data- and hypothesis-driven approach was chosen (Fig. 1): First, patients were stratified according to the Milan and UCSF criteria and the published cutoffs of the AFP model and Metroticket 2.0^14,16^. Second, mature miRNA profiling was performed on HCC tumor tissue from liver explants to quantify approximately 3600 miRNAs. This screening process included a log fold change (log₂FC) analysis. This analysis identified miRNAs with the greatest difference between recurrent and recurrence-free patients using predefined thresholds (log₂FC > 1 or < −1). Of the miRNAs that met the stringent significance criteria (*p* < 0.015), the one with the strongest discriminatory capacity was selected for further modeling. To improve discriminative performance and reduce model complexity by keeping the signature small, the remaining miRNAs were evaluated through a literature review. Only those with prior evidence in post-liver transplantation/HCC contexts were included in the model to develop a predictive miRNA signature based on combined discriminative expression levels. Third, the miRNA signature was combined with each clinical risk model to predict HCC recurrence and recurrence-free survival (RFS).

### Statistical analysis

Data are presented as total numbers and percentages, mean and standard deviation, or median and range, or 95% confidence interval (CI), as appropriate. Categorical data were compared using chi-squared tests, and t-test was used to compare two continuous variables. The Mann-Whitney test was used for nonparametric comparisons. Log_2_FC calculations were performed for miRNA expression analysis. A miRNA signature was constructed consisting of identified miRNAs that allowed classification of HCC recurrence vs. non-recurrence using multivariate logistic regression. A post-hoc power analysis was performed for each of the candidate miRNAs based on the observed between-group expression differences in the pilot cohort (recurrence vs. non-recurrence; *n* = 10 per group). Effect sizes were quantified using Cohen’s d, calculated from the group means and pooled standard deviations. Achieved statistical power was then estimated for a two-sided independent-samples t test at a significance level of α = 0.05. The performance of the clinical risk models alone and combined with the miRNA signature was evaluated using receiver operating characteristic (ROC) curve analysis. Internal validation was performed using leave-one-out cross-validation (LOOCV). For each iteration of LOOCV, the models were trained using data from 19 patients and then used to generate an out-of-sample predicted probability of recurrence for the patient held out. To prevent information leakage, miRNA predictors were z-standardized within each training fold, and the derived scaling parameters were applied to the held-out sample. Due to the small sample size and potential for (quasi-)separation, the clinical + miRNA and miRNA-only models were fitted using L2-penalized (ridge) logistic regression. Model discrimination was quantified using the area under the receiver operating characteristic curve (AUC). For the clinical-only models, discrimination was computed directly from the fixed binary clinical indicator (two-level score), which does not require refitting the model. Uncertainty was quantified using bootstrap resampling (5,000 resamples) to derive 95% confidence intervals (CIs) for the AUCs. The improvements in discrimination from adding miRNAs to each clinical model were summarized as ΔAUC, with paired bootstrap 95% confidence intervals (CIs) based on the same leave-one-out cross-validation (LOOCV) predictions. ROC curves were plotted for each clinical model and its corresponding clinical + miRNA model, including a reference diagonal. Patients were assigned to either a high-risk or low-risk group for HCC recurrence after LT according to a cutoff defined in the ROC analysis using Youden’s index. Kaplan-Meier survival analysis was used to estimate RFS, and the log-rank test was used to compare survival curves between the high-risk and low-risk groups^31^. Number-at-risk tables are displayed below the Kaplan–Meier curves. P values < 0.05 were considered statistically significant. Statistical analyses were performed with SPSS Statistics version 29 (SPSS Inc., Chicago, IL, USA), GraphPad Prism version 9.0 (GraphPad, San Diego, CA, USA) and R Studio version 2023.12.1 (Posit Software Inc., Boston, MA, USA).

## Results

### Baseline patient characteristics and imaging features

A total of 20 patients were included in the study and stratified according to confirmed HCC recurrence after LT or recurrence-free survival [recurrence: male, *n* = 10 (100%); median age at LT, 61.5 y; non-recurrence: male, *n* = 7 (70%); median age at LT, 65.0 y]. Baseline patient characteristics are summarized in Table 1.. The cause of liver disease was heterogeneous in both cohorts. In the recurrence cohort, the most common cause of liver disease was chronic alcohol abuse with subsequent alcoholic liver disease (40%), while in the non-recurrence cohort it was metabolic dysfunction-associated steatohepatitis (MASH; 50%). There were no patients with a history of bridging therapies in the recurrence cohort, which resulted in a difference in the chi-squared group comparison (*p* = 0.010). Otherwise, there were no significant differences between the two cohorts, indicating reasonable comparability.

**Table 1:** Baseline patient characteristics and imaging features

	Recurrence	Non-recurrence	P
Clinical and laboratory data			
N	10	10	
Sex			
Male	10 (100%)	7 (70%)	0.060
Female	0 (0%)	3 (30%)	
Age at liver transplantation (years), median (IQR)	61.5 (14.0)	65.0 (13.0)	0.693
Listing to transplant (days), median (IQR)	75.0 (251.0)	100.0 (331.0)	0.541
Etiology of liver disease			
Alcoholic	4 (40%)	2 (20%)	0.076
HBV	3 (30%)	2 (20%)	
HCV	3 (30%)	1 (10%)	
MASH	0 (0%)	5 (50%)	
History of bridging therapies	0 (0%)	5 (50%)	**0.010**
INR, median (IQR)	1.1 (0.4)	1.2 (0.2)	0.064
Bilirubin (mg/dL), median (IQR)	0.8 (1.9)	0.8 (0.8)	0.082
ALT (U/L), median (IQR)	44.5 (84.0)	40.0 (24.0)	0.357
AST (U/L), median (IQR)	99.5 (120.0)	51.5 (28.0)	**0.015**
ALP (U/L), median (IQR)	160.0 (103.0)	161.0 (99.0)	0.712
GGT (U/L), median (IQR)	107.5 (189.0)	139.0 (164.0)	0.240
AFP (U/L), median (IQR)	9.5 (38.3)	24.1 (280.6)	0.675
MELD, median (IQR)	9.0 (6.0)	9.0 (4.0)	0.132
APRI, median (IQR)	1.8 (3.6)	2.2 (11.4)	0.552
Imaging features			
Inside Milan	3 (30%)	4 (40%)	0.639
Irregular margin	8 (80%)	4 (40%)	0.068
Peritumoral enhancement	3 (30%)	1 (10%)	0.264

Abbreviations: AFP, Alpha fetoprotein; ALP, Alkaline phosphatase; ALT, Alanine transaminase; APRI, AST to Platelet Ratio Index; AST, Aspartate transaminase; CR, Complete response; GGT, Gamma-glutamyl transferase; HBV, Hepatitis B; HCV, Hepatitis C; INR, International normalized ratio; IQR, Interquartile range; MASH, Metabolic dysfunction-associated steatohepatitis; MELD, Model for End-Stage Liver Disease; PR, Partial response; SD, Standard deviation

### MiRNA expression analysis

**Fig. 2:**
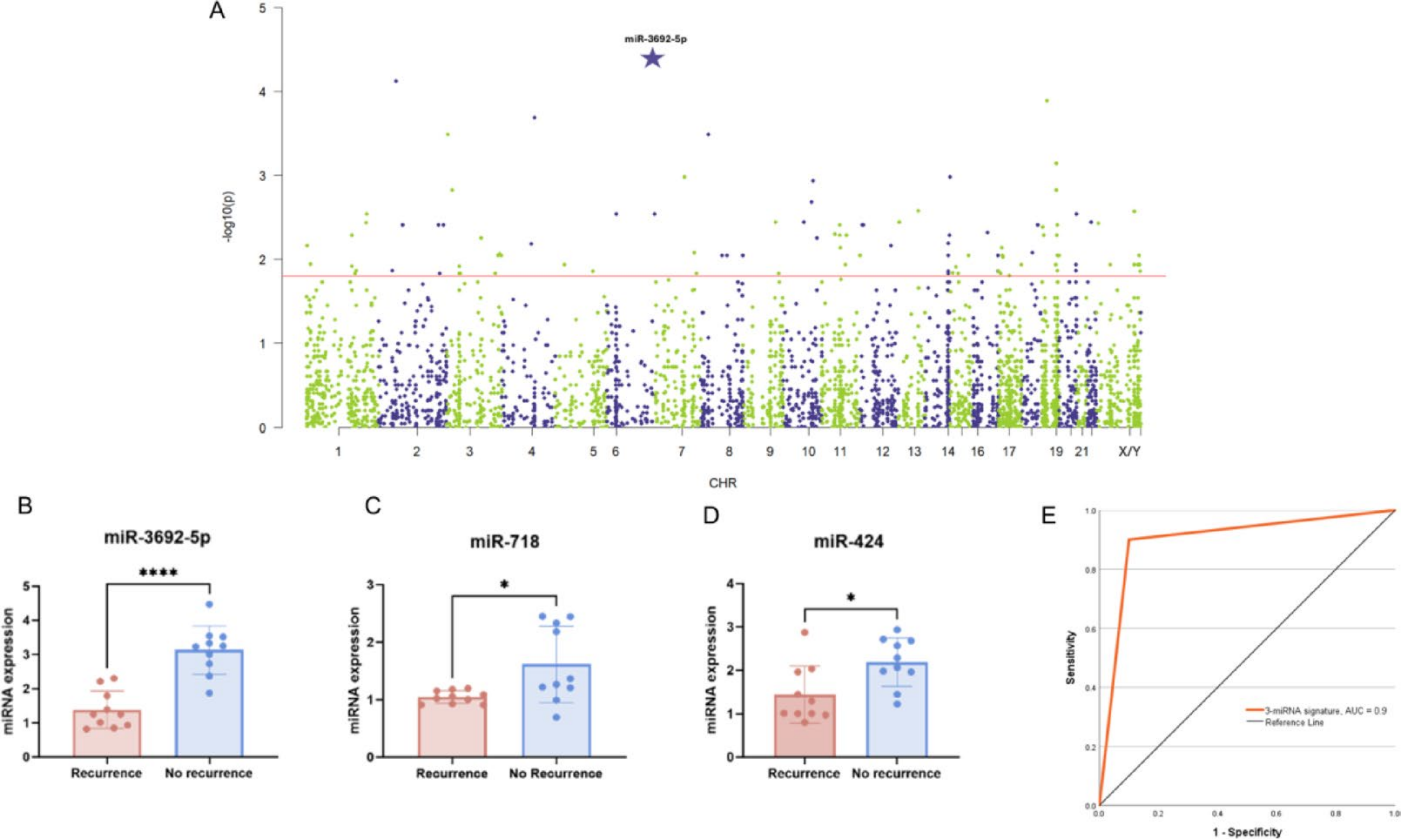
MiRNA profiling analysis. (**A**) Manhattan plot demonstrating differentially expressed miRNAs from FFPE HCC tissue between the recurrence and non-recurrence groups (red line, significance criterion *p* < 0.015). MiR-3692-5p (log_2_FC = 1.2) exhibited the strongest discriminatory capacity (asterisk). (**B**,** C** & **D**) Validation analysis of the data- and literature-driven miRNAs between patients with recurrence and non-recurrence for (**B**) miR-3692-5p, (**C**) miR-718, and (**D**) miR-424. (**E**) Performance of the 3-miRNA signature for predicting HCC recurrence using receiver operating characteristic (ROC) curve analysis.

Microarray profiling of approximately 3600 mature miRNAs in archived FFPE HCC tissues yielded 272 miRNAs meeting the log_2_FC threshold, of which 103 met the pre-specified significance criterion (*p* < 0.015, Fig. 2A red line) and were subjected to biomarker-oriented filtering for expression signal and reproducibility. MiR-3692-5p showed the strongest discriminatory capacity (Fig. 2A asterisk) with significant downregulation in the recurrence group (mean ± SD recurrence, 1.4 ± 0.5, non-recurrence, 3.1 ± 0.7; *p* < 0.0001, Fig. 2B) and was therefore retained as the primary data-driven candidate. Two additional miRNAs (miR-718 and miR-424) were included based on prior evidence in post-liver transplantation HCC cohorts (Table 2) and because they showed directionally consistent downregulation in the recurrence group in our dataset: miR-718 (mean ± SD recurrence, 1.0 ± 0.1, non-recurrence, 1.6 ± 0.7; *p* = 0.01, Fig. 2C); miR-424 (mean ± SD recurrence, 1.4 ± 0.7, non-recurrence, 2.2 ± 0.6; *p* = 0.01, Fig. 2D).

**Table 2:** Evidence supporting miR-718 and miR-424 in HCC and liver transplantation contexts.

miRNA	Cohort size (*n*)	Specimen/sample type	Assay platform	Directionality (comparison)	Key findings/results	Endpoint = post-LT recurrence	Reference
miR-718	Discovery: 6 (4 recurrence, 2 no recurrence); Validation: 59 (LDLT)	Preoperative serum exosomes (living-donor LT recipients)	Exosomal miRNA microarray (miRCURY LNA labeling + 3D-Gene Human miRNA Oligo chip, Toray) + qRT-PCR (TaqMan miRNA assays; LightCycler)	Recurrence vs. no recurrence: ↓	miR-718 significantly reduced in recurrence after LT; low miR-718 associated with aggressive tumor differentiation; functional data supported tumor-suppressive effect and implicated HOXB8 as a target axis.	Yes	^50^
miR-424	121 LT recipients (2003–2010); explant tumor + adjacent tissue	HCC tumor tissue and adjacent noncancerous liver from LT recipients (explant)	RT-qPCR (miScript II RT + miScript SYBR Green; ABI 7700; U6 control) + in vitro/in vivo functional assays	Tumor vs. adjacent: ↓; Recurrence vs. non-recurrence: ↓	Lower miR-424 in tumors vs. adjacent tissue and in recurrent vs. non-recurrent tumors; low miR-424 was an independent prognostic factor for recurrence and stratified recurrence-free survival, including in patients beyond Milan criteria.	Yes	^51^
miR-424	Microarray: 30 HCC samples; qRT-PCR: 96 paired HCC/ANLT; validation cohort: 70 paired fresh HCC/ANLT	HCC tumor tissue vs. adjacent non-tumorous liver tissue (non-LT surgical cohorts)	miRNA microarray profiling (840 mammalian miRNAs) + qRT-PCR validation; mechanistic assays (targets Akt3/E2F3; pathway reporter arrays)	HCC vs. ANLT: ↓	miR-424 frequently downregulated in HCC and inversely correlated with proliferation marker Ki-67; low miR-424 associated with adverse clinicopathologic features and worse OS/DFS; functional data support tumor-suppressive miR-424/Akt3/E2F3 axis.	No (OS/DFS, tumor features)	^52^
miR-424-5p	Liver tissues: 50 HCC resection patients; Sera: 62 HCC patients; Controls: 40 healthy	Anoikis-resistant/anchorage-deprived HCC cell models; human HCC tissues and sera (non-LT)	Microarray expression profiling (cells) + qRT-PCR (All-in-One miRNA qRT-PCR kit, GeneCopoeia; U6 control) + luciferase/functional assays	Anoikis-resistant vs. control cells: ↓; Metastatic vs. non-metastatic (tissue/sera): ↓	miR-424-5p decreased in anchorage-independent/anoikis-resistant HCC cells; restoration reversed anoikis resistance and EMT and reduced malignant behaviors; ICAT/CTNNBIP1 validated as a direct target; downregulated in HCC tissues/sera, especially in metastatic disease and higher grade/stage.	No (metastasis/grade/stage)	^40^

Abbreviations: LT, liver transplantation; LDLT, living-donor liver transplantation; ANLT, adjacent non-tumorous liver tissue; OS, overall survival; DFS, disease-free survival; NR, not reported.

A final combination of these 3 miRNAs provided the highest discriminative power for HCC recurrence versus non-recurrence, with an area under the receiver operating characteristic curve (AUC) of 0.9 (95% CI, 0.744–1, Fig. 2E). This 3-miRNA signature was subsequently used for risk stratification in conjunction with existing clinical models. The post-hoc power analysis **(**Table 3**)** revealed that miR-3692-5p had a substantial observed effect size (Cohen’s d = 2.79), indicating an achieved power greater than 99.9% with *n* = 10 per group. The effect sizes for miR-424 (Cohen’s d = 1.20) and miR-718 (Cohen’s d = 1.23) were also large, with achieved powers of 71.8% and 73.7%, respectively.

**Table 3:** Post-hoc power analysis.

miRNA	Mean Diff.	Cohen’s d	Observed Power (*n* = 10/group)
miR-3692-5p	1.7	2.79	> 99.9%
miR-424	0.6	1.20	71.8%
miR-718	0.8	1.23	73.7%

**Fig. 3:**
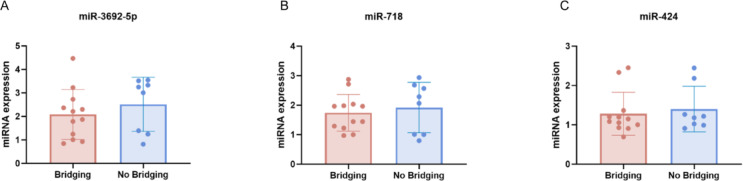
MiRNA expression analysis addressing potential confounding by bridging therapy. The expression levels of three key miRNAs - (**A**) miR-3692-5p, (**B**) miR-718, and (**C**) miR-424 - did not differ significantly between patients with and without a history of bridging therapy, regardless of recurrence status.

Additional analysis addressing potential confounding by bridging therapy: Expression of the three key miRNAs was compared between patients with and without a history of bridging therapy independent of recurrence status. There were no significant differences for miR-3692-5p (mean ± SD bridging, 2.1 ± 1.0 vs. no bridging, 2.5 ± 1.2; *p* = 0.40, Fig. 3A), miR-718 (1.7 ± 0.6 vs. 1.9 ± 0.9; *p* = 0.60, Fig. 3B), or miR-424 (1.3 ± 0.5 vs. 1.4 ± 0.6; *p* = 0.64, Fig. 3C).

### Integration of the miRNA signature with clinical risk models

**Fig. 4:**
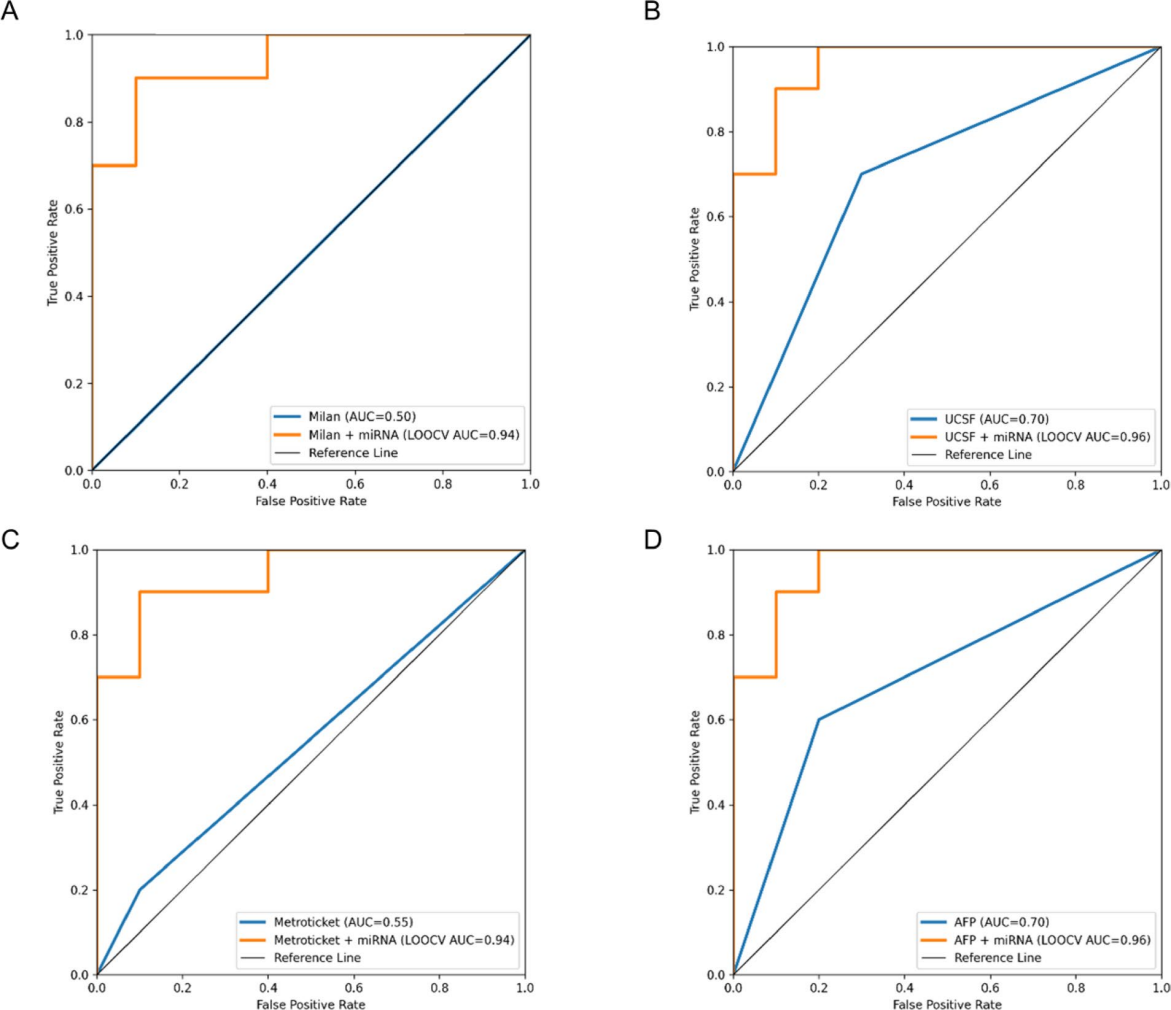
Performance of the stand-alone clinical models and after combination with the miRNA signature for predicting HCC recurrence using receiver operating characteristic (ROC) curve analysis. Abbreviations: AFP, Alpha-fetoprotein; UCSF; University of California San Francisco criteria.

All patients were evaluated using the Milan criteria, UCSF criteria, Metroticket 2.0 and the AFP model. When each clinical model was used alone, the AUCs for predicting HCC recurrence ranged from 0.5 to 0.7, with the UCSF criteria and the AFP model demonstrating the highest stand-alone predictive value (AUC, 0.7; 95% CI, 0.462–0.938, Fig. 4B **& D**). Across all four clinical frameworks, incorporating the three-miRNA signature produced high out-of-sample discriminatory performance in LOOCV:

- Milan criteria plus miRNA Signature: LOOCV-AUC 0.94 (95% CI 0.81–1.00), Fig. 4A.
- UCSF criteria plus miRNA Signature: LOOCV-AUC 0.96 (95% CI 0.86–1.00), Fig. 4B.
- Metroticket 2.0 plus miRNA Signature: LOOCV-AUC 0.94 (95% CI 0.81–1.00), Fig. 4C.
- AFP Model plus miRNA Signature: LOOCV-AUC 0.96 (95% CI 0.86–1.00), Fig. 4D.

Youden’s index was used to define optimal cutoffs for high- and low-risk classification with each combined model. Overall, the addition of miRNA profiling consistently reclassified 8 to 12 patients (40–60% of the cohort) from a lower to a higher risk category (or vice versa).

### Recurrence-free survival

**Fig. 5:**
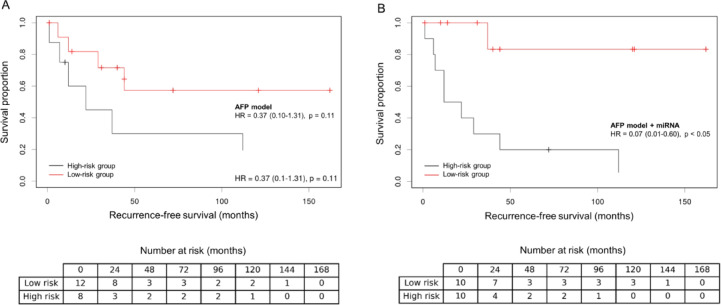
Analysis of recurrence-free survival of patients for the stand-alone AFP model (**A**) and after combined miRNA-based risk stratification (**B**). Abbreviations: AFP, Alpha-fetoprotein; HR, Hazard ratio.

Median follow-up was 51.0 months. Patients classified as high-risk by the combined models had a shorter estimated median RFS compared to those in the low-risk category. For example, when the miRNA signature was added to the AFP model, the high-risk group showed a median RFS of 17.0 months compared to 38.5 months in the low-risk group (HR = 0.07, *p* < 0.05; Fig. 5B) and discriminated better than the clinical model alone (*p* = 0.11; Fig. 5A). Similar stratification results were observed for the Milan and UCSF criteria and Metroticket 2.0 when augmented with the miRNA signature, all demonstrating significant separation of the RFS curves (each *p* < 0.05).

## Discussion

In this proof-of-concept study, we provide preliminary and hypothesis-generating evidence that integrating a tissue-derived miRNA signature with established clinical risk models may improve the prediction of HCC recurrence after liver transplantation. Our results indicate that the expression levels of three key miRNAs - miR-3692-5p, miR-424 and miR-718 - when combined with widely used criteria such as the Milan or UCSF criteria, Metroticket 2.0 and the AFP model, may improve the accuracy of recurrence prediction. Overall, the addition of miRNA profiling consistently reclassified 60% of patients in our cohort from a lower to a higher risk category (or vice versa), demonstrating a potentially meaningful shift in risk stratification. These results underscore the potential clinical value of incorporating molecular biomarkers into current HCC post-transplant risk stratification, advocating for more biologically nuanced approaches to post-transplant surveillance.

A notable aspect of our study was the downregulation of miR-3692-5p, miR-424, and miR-718 in HCC explants from patients who later experienced recurrence. It is widely accepted that miRNAs play a pivotal role in HCC development^32–36^. While the involvement of miRNAs in HCC pathogenesis has been extensively investigated, the precise function of miRNAs in HCC recurrence in transplanted livers remains largely elusive. In the present study, miR-3692-5p was identified as a potential predictor of HCC recurrence after LT in the largest cohort of human samples to date. Liu et al. demonstrated that miR-3692-5p plays a critical role in regulating the Warburg effect in HCC by modulating hypoxia-inducible factor (HIF-alpha)^37^. The upregulation of miR-3692-5p leads to the downregulation of HIF-alpha, thereby increasing the sensitivity to various therapies for HCC and thus may act as a preventive factor. Furthermore, Zhou et al. demonstrated that miR-3692-5p plays a pivotal role in a metabolic model comprising five major axes in HCC, as well as in the production of various signaling factors in HCC, highlighting the significance of this miRNA in HCC and its potential as a biomarker^38^. MiR-424 has been described as a preventive factor in HCC with an important role in anoikis and epithelial-mesenchymal transition (EMT), both processes involved in cancer metastasis and cell death^39,40^. Like miR-424, miR-718 has been described as a potentially preventive miRNA, with studies showing a positive correlation between miR-718 and the tumor suppressor phosphatase PTEN^41^. It has also been described as a negative regulator of early growth response protein 3 (EGR3) expression, thereby reducing the viability and proliferation of HCC cells^42^.

Our findings support and extend these observations by suggesting that such miRNAs not only reflect tumor biology, but may also refine post-transplant risk stratification beyond conventional imaging and tumor burden-based criteria. Furthermore, the fact that these miRNAs remained robust predictors even in archival FFPE tissue demonstrates the feasibility of incorporating miRNA assays into routine transplant surveillance protocols, especially as extraction and profiling methods continue to improve. This is consistent with emerging evidence from other studies highlighting the stability and reliability of miRNA as prognostic biomarkers in cancer and post-transplantation settings^43,44^.

From a clinical perspective, the use of size- and number-based criteria alone, such as Milan and UCSF, has been repeatedly criticized for overlooking the highly heterogeneous biological behavior of HCC^6,11,13^. Indeed, in our cohort, none of the patients who developed recurrence had received bridging therapies. In many centers, bridging or downstaging therapies are used to modulate tumor burden and improve transplant candidacy, and their role in altering tumor biology and recurrence patterns is the subject of ongoing investigation^45–47^. As described above, preclinical data indicate that miR-3692-5p directly targets hypoxia-inducible factor 1 alpha (HIF-1α) and restrains HIF-linked metabolic programs in HCC. Upstream suppression of miR-3692 can functionally de-repress HIF-1α signaling^37^. In contrast, bridging therapies like transarterial chemoembolization (TACE) intentionally induce ischemia and hypoxia, a microenvironmental shift expected to stabilize/activate HIF‑1α and promote adaptive, pro‑angiogenic signaling in residual tumor^48^. However, since post-TACE clinical miRNA studies have examined only a limited panel of circulating miRNAs and have shown time-dependent systemic miRNA shifts after TACE, the direction and magnitude of miRNA change in TACE-treated versus untreated HCC cannot be inferred without paired measurements (tumor and/or liquid biopsy)^49^. At least, we were unable to detect any differences in miRNA expression between the bridging and non-bridging cohorts, suggesting that the present bias can be mitigated to some extent.

Despite the promising findings, this study has several limitations that should be addressed in future research. First, the subgroups of patients investigated represent rare clinical conditions, making it challenging to obtain sufficient samples for specific analyses. This explains the relatively small cohort included in this proof-of-principle study. Overall, the pilot dataset was highly powered to detect the observed difference for miR-3692-5p, whereas modestly larger sample sizes would be expected to achieve ≥ 80% power for miR-424 and miR-718 in independent validation studies. Larger, multi-institutional cohorts will be essential to confirm and validate the proposed miRNA signature and to more thoroughly examine potential confounding factors. Second, the study utilized retrospectively collected samples with well-documented follow-up data. While this approach has value, a prospective study design, particularly one involving fresh-frozen material, would provide stronger evidence and reduce potential biases. Third, our analysis was based on tissue specimens. Although informative, future studies could benefit significantly from evaluating miRNA profiles in non-invasive sources such as serum or plasma. This approach would not only improve clinical applicability but also offer insights into the dynamic expression patterns and underlying pathophysiological processes. Finally, while our analysis identified specific miRNAs potentially involved in hepatocellular carcinogenesis, their exact functional roles remain to be elucidated. Future studies should explore their cellular functions and identify potential target genes to better understand their role in tumor biology. Additionally, the baseline imbalance in bridging therapy is a significant confounding factor that cannot be adequately addressed in this fixed cohort, which prevents reliable adjustment and causal attribution. Thus, the signature may capture both intrinsic tumor biology and treatment-modified biology. External validation in larger, prospectively characterized, multicenter cohorts with balanced treatment exposure is necessary.

In conclusion, our study demonstrates the feasibility and potential utility of integrating a miRNA signature into current HCC risk models to optimize post-transplant risk stratification and surveillance. The combination of miR-3692-5p, miR-424 and miR-718 was associated with improved ability of current imaging and AFP-based criteria to discriminate between patients at low and high risk of recurrence. Further studies are needed to identify objective molecular biomarkers that can support the management of HCC transplant patients in a more individualized and effective manner.

## Data Availability

The data that support the findings of this study are available from the corresponding author, P.S., upon reasonable request.
